# Age-related alterations in fatty acid metabolism: a clinical study of erythrocyte and plasma profiles in a population from Brandenburg, Germany

**DOI:** 10.3389/fragi.2026.1725187

**Published:** 2026-04-10

**Authors:** Yingqi Yu, Chaoxuan Wang, Ruirui Wang, Yifang Chen, Zeren Wei, Yanan Xiao, Ulf Elbelt, Anne Pietzner, Karsten H. Weylandt

**Affiliations:** 1 Medical Department B, Division of Hepatology, Gastroenterology, Oncology, Hematology, Palliative Care, Endocrinology and Diabetes, Brandenburg Medical School, University Hospital Ruppin-Brandenburg, Neuruppin, Germany; 2 Charité – Universitätsmedizin Berlin, Corporate member of Freie Universität Berlin and Humboldt-Universität zu Berlin, Campus Benjamin Franklin, Berlin, Germany; 3 Sichuan Provincial Key Laboratory for Human Disease Gene Study, Sichuan Provincial People’s Hospital, University of Electronic Science and Technology of China, Chengdu, China; 4 Department of Medicine, Pingxiang People’s Hospital, Gannan Medical University, Pingxiang, China; 5 MVZ Endokrinologikum Berlin, Berlin, Germany; 6 Faculty of Health Sciences, Joint Faculty of the Brandenburg University of Technology, Brandenburg Medical School and University of Potsdam, Potsdam, Germany

**Keywords:** aging, desaturase and elongase indices, fatty acids, omega-3, omega-6

## Abstract

**Introduction:**

Aging is accompanied by changes in lipid metabolism that may influence cellular homeostasis and risk for age-related disease. Circulating polyunsaturated fatty acid (PUFA) status is increasingly recognized as an important marker of metabolic health and may shift with age. Product-to-precursor ratios of fatty acids, including PUFA are commonly used as proxy indices of desaturation and elongation but do not directly reflect enzyme activity.

**Methods:**

In this cross-sectional study, plasma and erythrocyte fatty acid profiles were measured by gas chromatography–flame ionization detection (GC-FID) in patients (n = 1277) from a metabolic disease clinic in Brandenburg, Germany. Participants were stratified into five age groups (≤ 34, 35–44, 45–54, 55–64, ≥ 65 years) and differences between groups were assessed using statistical tests.

**Results:**

Participants aged ≥ 65 years had higher total omega-3 (n-3) and lower total omega-6 (n-6) PUFA levels in both matrices. Eicosapentaenoic acid (EPA) and docosahexaenoic acid (DHA) increased with age, whereas linoleic acid (LA) and dihomo-gamma-linolenic acid (DGLA) decreased. Ratio-based indices showed consistent age associations. The delta-5-desaturase index (D5D) and arachidonic acid (AA)/LA ratio were positively associated with age, while elongation of very long chain fatty acids (ELOVL)2 and ELOVL6 were inversely associated.

**Discussion:**

Overall, blood PUFA profiles and multiple ratio-based indices showed consistent, age-related trends in this clinical cohort. Interpretation is limited by the cross-sectional design and the lack of key determinants of PUFA status (e.g., diet, clinical covariates, genetic information and gut/microbiome factors). Nevertheless, these results underscore age-related shifts in PUFA composition and enzymatic proxy indices, providing new insights into lipid metabolism across the lifespan.

## 1 Introduction

Aging is an inherent biological phenomenon experienced by all living organisms, marked by a gradual deterioration in the body’s capacity to sustain homeostasis and mend damaged tissues. In humans, aging is intricate, shaped by a blend of genetic, environmental, and lifestyle factors (Dziechciaż and Filip, 2014). Amid the numerous elements impacting aging, fatty acid synthesis and metabolism assume a central role. Alterations in lipid metabolism contribute significantly to lifespan variation and the onset of age-related diseases (Hornburg et al., 2023). Consistent with the role of modifiable lifestyle factors in biological aging, a randomized controlled trial showed that vitamin D and omega-3 supplementation plus home-based exercise modestly slowed DNA methylation, based biological aging in older adults (Bischoff-Ferrari et al., 2025). These findings support a link between omega-3 (n-3) and omega-6 (n-6) status and biological aging, warranting further study of age-related blood polyunsaturated fatty acids (PUFA) patterns.

PUFA are essential components of cell membranes and precursors of important signaling molecules, with the n-3 and n-6 PUFA being the most prominent representatives (Harayama and Shimizu, 2020). As precursors for bioactive lipid metabolites, PUFA play important roles in human physiology and pathology (de Carvalho and Caramujo, 2018). N-3 PUFA, particularly docosahexaenoic acid (DHA) and eicosapentaenoic acid (EPA), are known for their competitive metabolic pathways with n-6 PUFA, particularly arachidonic acid (AA) (Schmitz and Ecker, 2008). This competition extends to the synthesis of many types of oxylipins, which are lipid mediators with profound effects on inflammation, thrombosis and atherosclerosis (Praticò and Dogné, 2009; Nayeem, 2018; Rabehl et al., 2024; Sherratt et al., 2024). The balance between n-6 and n-3 PUFA is critical and affects cell function, membrane fluidity, receptor signaling and gene expression (Schmitz and Ecker, 2008; Sunshine and Iruela-Arispe, 2017).

**FIGURE 1:**
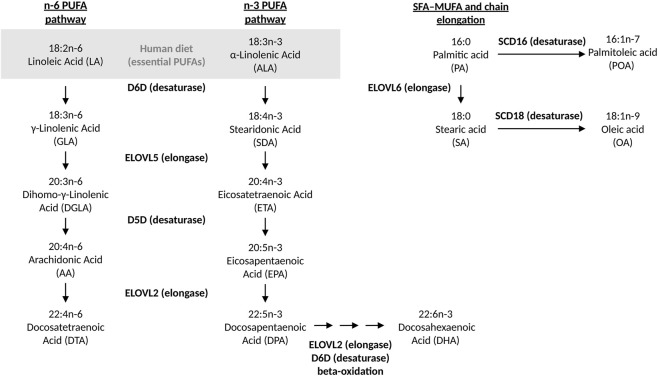
Overview of n-3, n-6, and SFA–MUFA metabolic pathways. The schematic shows the enzymes and fatty acids included in this study, with enzymatic activities represented by product-to-substrate ratios where applicable. Not all fatty acids depicted were analyzed, but the figure provides a comprehensive overview of the pathways investigated. Adapted from Rabehl et al. (2024).

The activity of enzymes involved in PUFA metabolism contributes to the regulation of lipid composition, product-to-precursor fatty acid ratios provide one means of gaining orientation on metabolic pathways (Li et al., 2013). They are used as indirect, ratio-based proxies of enzyme activity in human blood and tissues. However, they do not represent a direct enzymatic measurement and can be influenced by factors such as dietary fat intake, adiposity, inflammation and genetic variation (Warensjö et al., 2009; Lattka et al., 2010; Rabehl et al., 2024). Delta-5 desaturase (D5D) is a rate-limiting enzyme in PUFA biosynthesis that regulates the conversion of essential PUFA into long-chain PUFA (LC-PUFA). The D5D index is typically assessed as the ratio of arachidonic acid (AA) to dihomo-γ-linolenic acid (DGLA) and, in dietary contexts, reflects the body’s adaptive response to different types of fat intake (Vessby et al., 2013). Furthermore, the clinical significance of the D5D index lies in its ability to indicate the balance of PUFA within the body, which can be used to assess the risk of various metabolic disorders such as obesity, insulin resistance, and cardiovascular disease (Warensjö et al., 2009; Mousavi et al., 2021; Wang et al., 2022). Delta-6 Desaturase (D6D) catalyzes the conversion of linoleic acid (LA) to γ-linolenic acid (GLA) as well as additional steps in n-6 and n-3 PUFA biosynthesis. Few studies suggest that D6D activity may decline with age, contributing to altered fatty acid profiles and metabolic changes (Horrobin, 1981; Ali et al., 2023). Two additional desaturases, stearoyl CoA-desaturase (SCD)16 and SCD18, which convert saturated fatty acids to monounsaturated fatty acids by introducing a double bond, are also considered markers of metabolic health. Monounsaturated product-to-saturated precursor ratios can be used to indirectly estimate their enzymatic activity (Svendsen et al., 2020). Another group of enzymes involved in PUFA metabolism are the elongases, specifically Elongation of Very Long Chain Fatty Acids-Like (ELOVL2, ELOVL5, and ELOVL6, which catalyze the elongation of PUFA to LC-PUFA. In particular, DNA methylation at the ELOVL2 locus has been proposed as a robust epigenetic marker of chronological age (Garagnani et al., 2012; Paparazzo et al., 2023). A simplified scheme of n-6 and n-3 PUFA metabolic pathways highlighting the involved enzymes is shown in Figure 1.

Research characterizing age-related patterns of circulating fatty acids is limited, and longitudinal evidence describing within-individual changes in fatty acid profiles across aging remains scarce. Erythrocyte fatty acid composition is generally considered to reflect medium-term PUFA status over weeks to months, whereas plasma fatty acids are more sensitive to short-term dietary intake and metabolic fluctuations (Hodson et al., 2008; Yuzyuk et al., 2018; Parry et al., 2021). This study aimed to address this gap by examining age-related distributions of fatty acids in plasma and erythrocytes and by assessing indirect activity proxy indices of fatty acid-metabolizing enzymes, including D5D, D6D, ELOVL2, ELOVL5, SCD16, and SCD18. We further explored potential explanations for age-related differences in fatty acid profiles. Given the lack of detailed information on diet, body mass index (BMI), comorbidities and medication use, our analyses are interpreted as observational and not as direct evidence of altered enzymatic activity.

## 2 Materials and methods

### 2.1 Study population

We recruited adult patients who were admitted to the metabolic disease clinic (University Hospital Ruppin-Brandenburg, UKRB), Germany. The study was approved by the institutional ethics committee, and written informed consent was obtained from all subjects. Patients who did not provide written informed consent were excluded. In the analysis, patients (n = 1,277) were divided into five age groups: ≤34 years, 35–44 years, 45–54 years, 55–64 years, and ≥65 years. Age stratification was defined *a priori* based on previous epidemiological studies examining plasma fatty acids in relation to age, and on the age distribution of our clinical cohort (Diffenderfer et al., 2022; Ali et al., 2023). Patients’ blood samples were collected in ethylenediaminetetraacetic acid (EDTA) tubes and immediately centrifuged (3,500 rpm (2,800 × g), 4 °C, 10 min). Following centrifugation, plasma and erythrocytes were separated, the upper layer of plasma and the lower layer of erythrocytes were collected and stored at −80 °C until further analysis. The storage time at −80 °C between blood collection and gas chromatography flame-ionization detection (GC-FID) analysis was generally less than 1 week. The collection of erythrocyte samples started in November 2021 and concluded in September 2023, comprising 1,048 samples available for analysis. The collection of plasma samples started in December 2022 and concluded in September 2023, comprising 656 samples available for analysis. An overlapping subset of 427 participants with paired plasma and erythrocyte samples collected on the same day was analyzed separately to complete the analytical assessment (n = 427) (Supplementary Tables S2A, S2B).

### 2.2 Sample preparation

Each sample was analyzed by GC-FID using 100 µL of erythrocytes and 75 µL of plasma. Fatty acid methylation and extraction were performed using adapted established protocols (Kang and Wang, 2005; Ostermann et al., 2014; Wang et al., 2022). Initially, samples were thawed at room temperature (RT), and both plasma and erythrocyte samples were mixed with 500 µL of boron trifluoride (BF_3_, Sigma-Aldrich Chemie GmbH, Taufkirchen, Germany) in 14% methanol and 500 µL of *n*-hexane (both from Merck KGaA, Darmstadt, Germany). The samples were incubated at 100 °C for 60 min in a preheated heating block. Samples were then cooled to RT, and 750 µL of water were added to facilitate phase separation. After centrifugation (3,500 rpm (1,300 × g), 4 °C, 5 min) 100 µL of the upper *n*-hexane layer was transferred into a micro-insert within a GC-FID glass vial for subsequent GC-FID analysis.

### 2.3 Determination of fatty acids using GC-FID

GC-FID analysis was conducted using a 7890B GC-FID System from Agilent Technologies, Santa Clara, CA, United States. This system was equipped with an HP-88 column (dimensions: 60 m × 0.25 mm × 0.2 µm, Agilent Technologies). The temperature settings varied as follows: initial temperature of 50 °C, ramped to 150 °C at a rate of 20 °C/min, then to 240 °C at 6 °C/min, maintaining at 240 °C for 10 min, resulting in a total run time of 30 min. Nitrogen served as the carrier gas, maintained at a steady flow of 1 mL/min. For the sample injection, 1 µL was injected via splitless injection at 280 °C. FID operated at 250 °C with gas flows set at 20 mL/min for hydrogen, 400 mL/min for air, and 25 mL/min for makeup gas. Identification of methylated fatty acids in the samples was achieved by comparing retention times with a standard reference mix, the Supelco® 37 FAME MIX standard (CRM47885, Sigma-Aldrich, Laramie, WY, United States) and single methylated fatty acid standards (docosapentaenoic acid methyl ester, cis-vaccenic acid methyl ester, all-cis-4,7,10,13,16-docosapentaenoic acid methyl ester, cis-7,10,13,16-docosatetraenoic acid methyl ester (all single lipid standards were purchased from Cayman Chemical, Ann Arbor, MI, United States). Peak analyses and integration were performed using OpenLAB CDS ChemStation Edition software from Agilent Technologies. Erythrocyte and plasma fatty acids were quantified by GC-FID using pentadecanoic acid (C15:0) as the internal standard (50 μL, 1 μg/μL, added prior to methylation). Results in the main text are expressed as relative composition (%), whereas participant-level absolute plasma fatty acid concentrations are expressed as (µg/mL), calculated by the internal-standard method (provided in Supplementary Table S1).

Product-to-precursor fatty acid ratios were calculated as estimated desaturase/elongase indices (proxies that do not directly measure enzyme activity). D5D was defined as AA/DGLA (20:4n-6/20:3n-6) (Kröger et al., 2011). D6D was estimated primarily as GLA/LA (18:3n-6/18:2n-6) (Kröger et al., 2011), additionally, DGLA/LA (20:3n-6/18:2n-6) and AA/LA (20:4n-6/18:2n-6) were calculated as more distal composite n-6 proxies (Vessby et al., 2013). SCD-indices were SCD16, defined as palmitoleic acid (POA)/palmitic acid (PA) (16:1n-7/16:0) and SCD18, defined as oleic acid (OA)/stearic acid (SA) (18:1n-9/18:0) (Warensjö et al., 2008). Elongation indices included ELOVL5, defined as DGLA/GLA (20:3n-6/18:3n-6) and ELOVL6, defined as SA/PA (18:0/16:0) (Drąg et al., 2017). ELOVL2-related elongation proxies were docosatetraenoic acid (DTA)/AA (22:4n-6/20:4n-6) (Cormier et al., 2014), n-3 docosapentaenoic acid (DPA)/EPA (22:5n-3/20:5n-3) (Moussa et al., 2019), which reflect specific ELOVL2-mediated elongation steps, as well as DHA/DPA (22:6n-3/22:5n-3) (Borges et al., 2022), which serves as a more general marker of the overall metabolic conversion of DPA to DHA, including ELOVL2-dependent elongation.

Fatty acid classes were calculated as the sum (Σ) of the respective individual fatty acids identified in the analysis. Specifically for erythrocytes, ΣSFA comprised myristic acid (14:0), PA (16:0), SA (18:0), arachidic acid (20:0), behenic acid (22:0), and lignoceric acid (24:0). ΣMUFA included POA (16:1n-7), OA (18:1n-9), gondoic acid (20:1n-9), and nervonic acid (24:1n-9). ΣPUFA included alpha-linolenic acid (ALA, 18:3n-3), EPA (20:5n-3), DPA (22:5n-3), DHA (22:6n-3), LA (18:2n-6), GLA (18:3n-6), eicosadienoic acid (20:2n-6), DGLA (20:3n-6), AA (20:4n-6), DTA (22:4n-6), and n-6 docosapentaenoic acid (22:5n-6). Σn-3 PUFA included ALA (18:3n-3), EPA (20:5n-3), DPA (22:5n-3), and DHA (22:6n-3). Σn-6 PUFA included LA (18:2n-6), GLA (18:3n-6), eicosadienoic acid (20:2n-6), DGLA (20:3n-6), AA (20:4n-6), DTA (22:4n-6), and n-6 docosapentaenoic acid (22:5n-6). For plasma, ΣSFA additionally included lauric acid (12:0), tridecanoic acid (13:0), heneicosanoic acid (21:0), and tricosanoic acid (23:0). ΣMUFA additionally included myristoleic acid (14:1n-5), heptadecenoic acid (17:1n-7), and elaidic acid (18:1n-9). ΣPUFA additionally included mead acid (20:3n-9) and docosadienoic acid (22:2n-6) and Σn-6 PUFA additionally included docosadienoic acid (22:2n-6).

### 2.4 Statistical analysis

Erythrocyte and plasma datasets were analyzed independently, the overlapping subgroup with paired measurements was analyzed separately using the same procedures to assess consistency and comparability of results. Fatty acids were analyzed as relative composition (% of total fatty acids) in both matrices, for plasma, absolute concentrations (µg/mL) were additionally analyzed using the same statistical approach. Group comparisons across five age categories (≤34, 35–44, 45–54, 55–64, and ≥65 years) were performed using the Kruskal–Wallis test, with planned *post hoc* contrasts versus the ≥65-year reference group using Dunn’s multiple comparisons test with multiplicity-adjusted p values. Descriptive statistics are presented as mean ± SD for comparability. Associations with age were assessed using Spearman’s rank correlation (two-tailed, complete-case) and linear regression with age as a continuous predictor (unadjusted). Sex was available for all participants but was not included as a covariate in the regression models, residual confounding by sex cannot be excluded. No imputation was performed, and all measurements passing internal quality-control checks were retained. Analyses were performed in GraphPad Prism (version 10.2.3).

## 3 Results

### 3.1 Age-related changes in fatty acid distribution

To evaluate age-related differences in blood fatty acid profiles, participants were categorized into five age groups (≤34, 35–44, 45–54, 55–64, and ≥65 years). Age-group distributions are shown in Table 1, additional covariates were not available for multivariable adjustment. Blood fatty acid profiles across age groups are summarized in Table 2 (erythrocytes) and Table 3 (plasma) and Supplementary Tables S2A, S2B for the overlapping group. Because key clinical and lifestyle covariates (e.g., diet, BMI, comorbidities and medication use) were not consistently available, the following results should be interpreted as observational associations.

**TABLE 1:** Age group distribution of participants in the plasma and erythrocyte sub-studies.

Age group (years)	Erythrocytes (n = 1,048)	Plasma (n = 656)	Overlap (n = 427)
≤34	25.2 ± 4.2 (n = 111) Female: 60 (54.1%)	27.9 ± 4.7 (n = 34) Female: 17 (50.0%)	27.8 ± 4.4 (n = 23) Female: 11 (47.8%)
35–44	39.6 ± 2.9 (n = 115) Female: 45 (39.1%)	39.5 ± 2.8 (n = 74) Female: 33 (44.6%)	39.1 ± 2.8 (n = 53) Female: 24 (45.3%)
45–54	50.4 ± 2.7 (n = 174) Female: 80 (46.0%)	50.1 ± 2.8 (n = 108) Female: 53 (49.1%)	50.2 ± 2.6 (n = 67) Female: 34 (50.7%)
55–64	60.0 ± 2.8 (n = 302) Female: 145 (48.0%)	60.0 ± 3.0 (n = 212) Female: 98 (46.2%)	60.2 ± 2.9 (n = 130) Female: 64 (49.2%)
≥65	72.4 ± 6.2 (n = 346) Female: 140 (40.5%)	72.3 ± 6.0 (n = 228) Female: 97 (42.5%)	72.2 ± 6.2 (n = 154) Female: 64 (41.6%)

Data are shown as mean age ±SD, with sample size (n) for each age group. The number and percentage of female participants are reported for each dataset. The overlap column indicates participants with both erythrocyte and plasma measurements (total overlap n = 427).

**TABLE 2:** Erythrocyte fatty acid composition across age groups.

Age group (n)	ΣSFA	ΣMUFA	ΣPUFA	Σn-3 PUFA	Σn-6 PUFA	EPA + DHA
≤34 (n = 111)	45.1 ± 2.3**	18.3 ± 1.6	36.5 ± 2.2***	7.3 ± 1.4***	29.2 ± 2.2***	4.7 ± 1.1***
35–44 (n = 115)	45.6 ± 3.1	18.5 ± 2.4	35.8 ± 2.8	7.8 ± 1.9***	28.0 ± 2.3***	5.1 ± 1.6***
45–54 (n = 174)	45.5 ± 2.3	18.7 ± 1.9	35.8 ± 2.6	7.9 ± 1.7***	27.9 ± 2.4***	5.4 ± 1.3***
55–64 (n = 302)	45.8 ± 2.7	19.3 ± 3.1	34.9 ± 2.7	8.2 ± 1.8**	26.7 ± 2.5	5.6 ± 1.4***
≥65 (n = 346)	45.9 ± 2.4	18.8 ± 2.3	35.4 ± 2.6	8.7 ± 1.8	26.7 ± 2.3	6.0 ± 1.5
Overall p	0.011	0.021	<0.001	<0.001	<0.001	<0.001

Values are presented as mean ± SD (% of total fatty acids). ΣSFA, ΣMUFA, and ΣPUFA, denote the sums of saturated, monounsaturated and polyunsaturated fatty acids, respectively. Overall p were obtained using the Kruskal–Wallis test across five age groups. Post hoc comparisons were performed using Dunn’s multiple comparisons test with the ≥65-year group as the reference; asterisks indicate significant differences versus ≥65 years (*p < 0.05, **p < 0.01, ***p < 0.001; adjusted).

**TABLE 3:** Plasma fatty acid composition across age groups.

Age group (n)	ΣSFA	ΣMUFA	ΣPUFA	Σn-3 PUFA	Σn-6 PUFA	EPA + DHA
≤34 (n = 34)	39.1 ± 3.4	24.5 ± 3.6	36.5 ± 3.4	4.1 ± 1.0**	32.3 ± 3.1**	2.8 ± 0.9**
35–44 (n = 74)	38.5 ± 3.5	25.2 ± 4.4	36.3 ± 4.1	4.0 ± 1.5***	32.3 ± 3.9***	2.8 ± 1.3***
45–54 (n = 108)	38.5 ± 3.1	26.2 ± 3.8	35.4 ± 3.9	4.6 ± 1.4**	30.7 ± 3.8	3.2 ± 1.3**
55–64 (n = 212)	38.6 ± 3.0	26.2 ± 4.4	35.2 ± 4.5	4.8 ± 1.5	30.3 ± 4.2	3.5 ± 1.3
≥65 (n = 228)	38.7 ± 3.6	26.2 ± 4.3	35.2 ± 4.0	5.1 ± 1.7	30.0 ± 3.7	3.8 ± 1.5
Overall p	0.947	0.061	0.101	<0.001	<0.001	<0.001

Values are presented as mean ± SD (% of total fatty acids). Overall p were obtained using the Kruskal–Wallis test across five age groups. Post hoc comparisons were performed using Dunn’s multiple comparisons test with the ≥65-year group as the reference; asterisks indicate significant differences versus ≥65 years (*p < 0.05, **p < 0.01, ***p < 0.001; adjusted).

In erythrocytes (Table 2), sum(Σ)SFA was lower in the ≤34-year group compared with the ≥65-year group, while ΣPUFA was higher. ΣMUFA showed a small overall difference across age groups (Kruskal–Wallis p = 0.021) but no Dunn-adjusted differences versus the ≥65-year reference group. Σn-3 PUFA increased across age categories, with all younger groups showing lower Σn-3 PUFA than the ≥65-year group. In contrast, Σn-6 PUFA decreased with advancing age, with the ≤34, 35–44, and 45–54-year groups showing higher Σn-6 PUFA than the ≥65-year group. The sum of erythrocyte EPA + DHA (% of total fatty acids), also referred to as the Omega-3 Index (O3I) according to Harris and von Schacky (Harris and Von Schacky, 2004), was lower in younger age groups than in the ≥65-year group.

In plasma (Table 3), ΣSFA, ΣMUFA, and ΣPUFA did not differ across age groups. Absolute plasma concentrations (µg/mL) for all fatty acids are provided in Supplementary Table S1. Σn-3 PUFA differed across age groups and was lower in the ≤34, 35–44, and 45–54-year age groups compared with the ≥65-year group. EPA + DHA increased across age categories, mirroring the erythrocyte findings. Σn-6 PUFA also differed across age groups, with the ≤34 and 35–44 groups showing higher Σn-6 PUFA than the ≥65-year group.

### 3.2 Individual n-3 PUFA in different age groups

**FIGURE 2:**
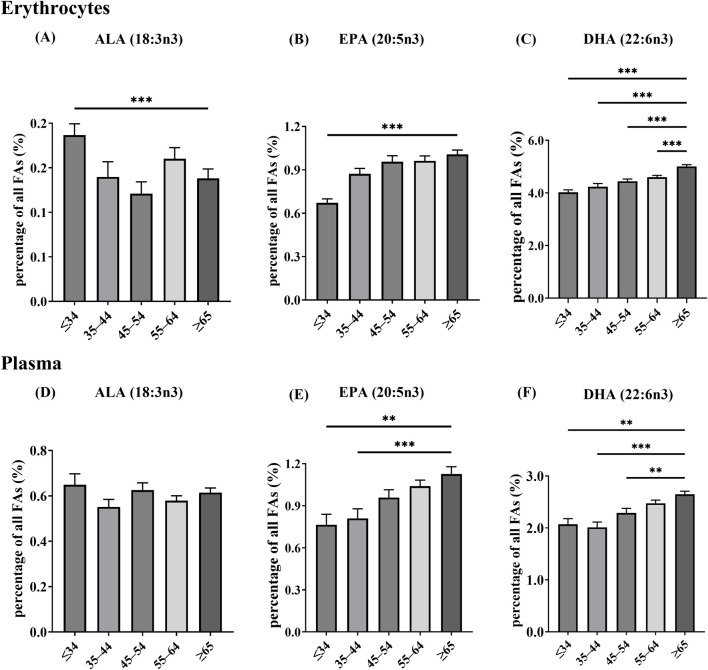
The distribution of blood n-3 PUFA levels across various age groups, values are shown as mean ± SD. **(A)** ALA in erythrocytes; **(B)** EPA in erythrocytes; **(C)** DHA in erythrocytes; **(D)** ALA in plasma; **(E)** EPA in plasma; **(F)** DHA in plasma. Erythrocytes, age groups: ≤34 years (n = 111); 35–44 (n = 115); 45–54 (n = 174); 55–64 (n = 302); ≥65 (n = 346); total (n = 1,048). Plasma, age groups: ≤34 years (n = 34); 35–44 (n = 74); 45–54 (n = 108); 55–64 (n = 212); ≥65 (n = 228); total (n = 656). *p < 0.05, **p < 0.01, ***p < 0.001. Asterisks indicate Dunn-adjusted *post hoc* differences versus the ≥65-year group.

We analyzed age-group differences in individual n-3 PUFA levels to characterize age-related variation in fatty acid profiles. In erythrocytes, ALA differed across age categories, with the ≥65-year group showing lower ALA than the ≤34-year group (multiplicity-adjusted p < 0.001), whereas no other age group differed from the ≥65-year group after multiplicity correction (Figure 2A). Erythrocyte EPA also varied by age, compared with the ≥65-year group, only the ≤34-year group had significantly lower EPA after multiplicity correction (p < 0.001) (Figure 2B). In contrast, erythrocyte DHA showed a clear age-related gradient: DHA was significantly lower in the ≤34, 35–44, 45–54, and 55–64-year age groups than in the ≥65-year group (all multiplicity-adjusted p < 0.001), indicating progressively higher erythrocyte DHA with advancing age (Figure 2C).

In plasma, ALA did not differ significantly across age categories (Figure 2D). Plasma EPA and DHA increased with age, with younger participants showing lower levels than those aged ≥65 years. Specifically, plasma EPA was significantly lower in the ≤34 and 35–44- year age groups compared with the ≥65-year group (multiplicity-adjusted p < 0.01 and p < 0.001, respectively) (Figure 2E). Plasma DHA was significantly lower in the ≤34, 35–44, and 45–54-year age groups than in the ≥65-year group (multiplicity-adjusted p < 0.01, p < 0.001, and p < 0.01, respectively), whereas the 55–64-year group did not differ significantly from the ≥65-year group after multiplicity correction (Figure 2F).

### 3.3 Individual n-6 PUFA in different age groups

**FIGURE 3:**
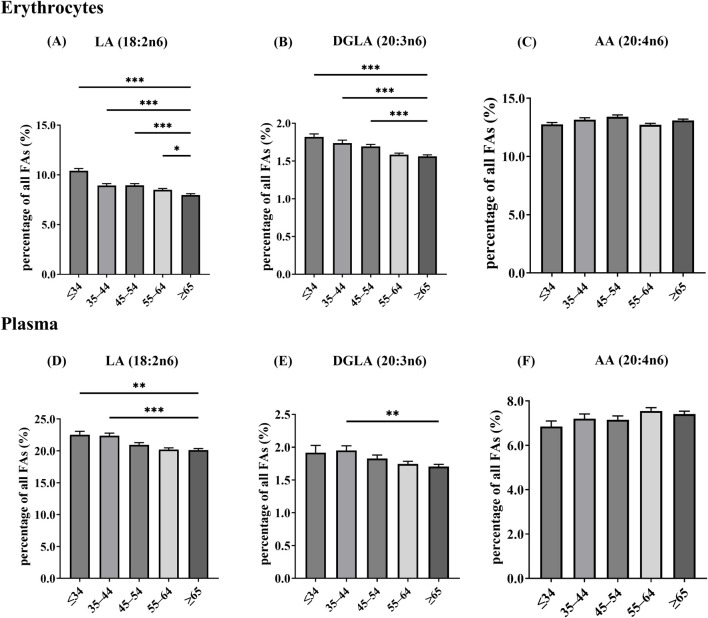
The distribution of blood n-6 PUFA levels across various age groups, values are shown as mean ± SD. **(A)** LA in erythrocytes; **(B)** DGLA in erythrocytes; **(C)** AA in erythrocytes; **(D)** LA in plasma; **(E)** DGLA in plasma; **(F)** AA in plasma. Erythrocytes, age groups: ≤34 years (n = 111); 35–44 (n = 115); 45–54 (n = 174); 55–64 (n = 302); ≥65 (n = 346); total (n = 1,048). Plasma, age groups: ≤34 years (n = 34); 35–44 (n = 74); 45–54 (n = 108); 55–64 (n = 212); ≥65 (n = 228); total (n = 656). *p < 0.05, **p < 0.01, ***p < 0.001. Asterisks indicate Dunn-adjusted *post hoc* differences versus the ≥65-year group.

To investigate the age-specific differences in the n-6 PUFA metabolism, we examined erythrocyte and plasma levels of LA and its metabolites DGLA and AA, respectively. The erythrocyte results showed that compared to individuals ≥65 years, individuals ≤34 years, as well as those in the age groups of 35–44, 45–54 and 55–64 years, exhibited significantly higher levels of LA (p < 0.001, p < 0.001, p < 0.001, and p < 0.05, respectively) (Figure 3A). DGLA levels were significantly higher in individuals 34, 35–44 and 45–54 years old compared to those ≥65 years (p < 0.001, p < 0.001, and p < 0.001, respectively) (Figure 3B). However, it is noteworthy that AA did not show any significant variation across the different age groups (Figure 3C). Plasma results showed similar trends, older adults (≥65 years old) had significantly lower LA than individuals ≤34 and 35–44 years old (p < 0.01 and p < 0.001, respectively) (Figure 3D). DGLA was also significantly lower in the elderly compared to individuals between 35 and 44 years old (p < 0.01) (Figure 3E). Plasma AA did not show significant differences across age groups after multiplicity adjustment (Figure 3F).

### 3.4 Age-related variation in desaturase and elongase indices

In addition to individual fatty acids, we calculated predefined ratio-based proxy indices of desaturation and elongation (D5D, D6D proxies, SCD16, SCD18, and ELOVL2/5/6-related indices) in erythrocytes and plasma. Index values across five age categories and associations with age as a continuous variable are summarized in Table 4.

**TABLE 4:** Age-stratified proxy indices and associations with age in erythrocytes (A) and plasma (B).

Index	Age groups (mean ± SD)					Overall p	Spearman			Linear regression	
	≤34 (n = 111)	35–44 (n = 115)	45–54 (n = 174)	55–64 (n = 302)	≥65 (n = 346)		n	ρ (95% CI)	p	Slope (95% CI)	p/R^2^
A. Erythrocytes											
D5D (AA/DGLA)	7.4 ± 2.0***	8.0 ± 2.0*	8.2 ± 2.1	8.5 ± 2.5	8.8 ± 2.6 (n = 345)	<0.001	1,047	0.162 (0.101–0.222)	<0.001	2.831e-02 (1.915e-02 to 3.747e-02)	<0.001 0.034
D6D (GLA/LA)	0.012 ± 0.011** (n = 110)	0.009 ± 0.016 (n = 112)	0.010 ± 0.022 (n = 169)	0.011 ± 0.018 (n = 295)	0.013 ± 0.026 (n = 331)	<0.001	1,017	−0.036 (−0.099 to 0.027)	0.252	5.000e-05 (−3.212e-05 to 1.322e-04)	0.232 0.001
D6D (DGLA/LA)	0.178 ± 0.049* (n = 110)	0.194 ± 0.060 (n = 112)	0.187 ± 0.046 (n = 169)	0.188 ± 0.053 (n = 295)	0.205 ± 0.220 (n = 331)	0.151	1,017	0.061 (−0.002–0.124)	0.051	5.500e-04 (3.110e-05 to 1.064e-03)	0.038 0.004
AA/LA	1.27 ± 0.34*** (n = 110)	1.47 ± 0.31** (n = 112)	1.52 ± 0.40* (n = 169)	1.54 ± 0.46* (n = 295)	1.81 ± 2.93 (n = 331)	<0.001	1,017	0.231 (0.171–0.290)	<0.001	1.163e-02 (4.989e-03 to 1.827e-02)	<0.001 0.011
ELOVL2 (DTA/AA)	0.253 ± 0.076**	0.238 ± 0.101	0.217 ± 0.088	0.229 ± 0.099	0.224 ± 0.084	0.009	1,048	−0.091 (−0.153 to −0.029)	0.003	−5.600e-04 (−9.066e-04 to −2.096e-04)	0.002 0.009
ELOVL2 (DHA/DPA)	1.696 ± 0.510*** (n = 109)	1.698 ± 0.880*** (n = 109)	1.925 ± 1.091** (n = 161)	2.006 ± 1.310 (n = 288)	1.984 ± 0.866 (n = 329)	<0.001	996	0.187 (0.124–0.248)	<0.001	6.630e-03 (2.606e-03 to 1.065e-02)	0.001 0.010
ELOVL2 (DPA/EPA)	4.038 ± 1.494***	3.348 ± 1.508	3.052 ± 1.712	3.059 ± 1.461 (n = 301)	3.065 ± 1.513	<0.001	1,047	−0.144 (−0.204 to −0.082)	<0.001	−1.581e-02 (−2.174e-02 to −9.875e-03)	<0.001 0.026
ELOVL5 (DGLA/GLA)	11.9 ± 4.8*** (n = 78)	8.0 ± 4.3 (n = 41)	9.4 ± 5.6* (n = 61)	7.6 ± 3.6 (n = 120)	7.3 ± 4.2 (n = 136)	<0.001	436	−0.273 (−0.360 to −0.181)	<0.001	−8.062e-02 (−1.045e-01 to −5.670e-02)	<0.001 0.092
ELOVL6 (SA/PA)	0.760 ± 0.096	0.757 ± 0.091	0.755 ± 0.089	0.734 ± 0.102	0.737 ± 0.093	0.039	1,048	−0.111 (−0.172 to −0.049)	<0.001	−7.100e-04 (−1.073e-03 to −3.411e-04)	<0.001 0.014
SCD16 (POA/PA)	0.023 ± 0.013**	0.025 ± 0.012	0.028 ± 0.014	0.031 ± 0.023***	0.026 ± 0.014	<0.001	1,048	0.046 (−0.017–0.108)	0.139	5.000e-05 (−1.613e-05 to 1.143e-04)	0.140 0.002
SCD18 (OA/SA)	0.814 ± 0.141	0.798 ± 0.172	0.817 ± 0.117	0.843 ± 0.234	0.824 ± 0.165	0.213	1,048	0.049 (−0.013–0.111)	0.113	6.100e-04 (−8.168e-05 to 1.306e-03)	0.084 0.003

Index	Age groups (mean ± SD)					Overall p	Spearman			Linear regression	
	≤34 (n = 34)	35–44 (n = 74)	45–54 (n = 108)	55–64 (n = 212)	≥65 (n = 228)		n	ρ (95% CI)	p	Slope (95% CI)	p/R2
B. Plasma											
D5D (AA/DGLA)	3.7 ± 1.4** (n = 33)	3.9 ± 1.4** (n = 73)	4.3 ± 1.6	4.4 ± 1.7 (n = 205)	4.5 ± 1.6 (n = 224)	<0.001	643	0.157 (0.078–0.233)	<0.001	1.763e-02 (8.465e-03 to 2.680e-02)	<0.001 0.022
D6D (GLA/LA)	0.0176 ± 0.0100	0.0183 ± 0.0106	0.0212 ± 0.0124	0.0207 ± 0.0122	0.0193 ± 0.0106	0.337	656	0.011 (−0.068–0.090)	0.776	2.000e-05 (−4.927e-05 to 7.949e-05)	0.645 0.000
D6D (DGLA/LA)	0.0879 ± 0.0371	0.0903 ± 0.0390	0.0905 ± 0.0340	0.0903 ± 0.0378	0.0887 ± 0.0349	0.976	656	−0.010 (−0.089 to 0.069)	0.798	−1.000e-05 (−2.099e-04 to 1.969e-04)	0.950 0.000
AA/LA	0.31 ± 0.09*	0.33 ± 0.11*	0.36 ± 0.14	0.40 ± 0.17	0.39 ± 0.16	0.002	656	0.117 (0.039–0.194)	0.003	1.580e-03 (7.198e-04 to 2.434e-03)	<0.001 0.020
ELOVL2 (DTA/AA)	0.0562 ± 0.0617	0.0343 ± 0.0364	0.0394 ± 0.0394	0.0355 ± 0.0358	0.0391 ± 0.0444	0.605	656	−0.006 (−0.084 to 0.073)	0.884	−1.000e-04 (−3.290e-04 to 1.354e-04)	0.413 0.001
ELOVL2 (DHA/DPA)	3.2 ± 1.1 (n = 31)	3.1 ± 1.5*** (n = 68)	3.5 ± 1.4	3.6 ± 1.2 (n = 209)	3.7 ± 1.2 (n = 219)	<0.001	635	0.151 (0.071–0.228)	<0.001	1.261e-02 (5.279e-03 to 1.995e-02)	0.001 0.018
ELOVL2 (DPA/EPA)	0.874 ± 0.405 (n = 31)	0.845 ± 0.386* (n = 66)	0.813 ± 0.334 (n = 105)	0.776 ± 0.362 (n = 203)	0.777 ± 0.471 (n = 223)	0.017	628	−0.117 (−0.196 to −0.037)	0.003	−2.160e-03 (−4.503e-03 to 1.854e-04)	0.071 0.005
ELOVL5 (DGLA/GLA)	4.95 ± 2.43 (n = 30)	4.98 ± 2.35 (n = 65)	4.53 ± 2.18 (n = 100)	4.48 ± 2.11 (n = 193)	4.61 ± 2.13 (n = 208)	0.531	596	−0.030 (−0.112 to 0.053)	0.464	−6.220e-03 (−1.909e-02 to 6.642e-03)	0.343 0.002
ELOVL6 (SA/PA)	0.362 ± 0.059	0.371 ± 0.061	0.361 ± 0.052	0.367 ± 0.060	0.358 ± 0.058	0.181	656	−0.102 (−0.179 to −0.023)	0.009	−3.600e-04 (−6.900e-04 to −3.745e-05)	0.029 0.007
SCD16 (POA/PA)	0.070 ± 0.026	0.074 ± 0.028	0.086 ± 0.036	0.090 ± 0.042*	0.080 ± 0.038	0.002	656	0.003 (−0.076–0.082)	0.936	1.100e-04 (−1.042e-04 to 3.246e-04)	0.313 0.002
SCD18 (OA/SA)	2.10 ± 0.62	2.18 ± 0.69	2.28 ± 0.64	2.27 ± 0.70	2.33 ± 0.74	0.267	656	0.085 (0.006–0.163)	0.030	4.870e-03 (9.705e-04 to 8.779e-03)	0.015 0.009

Values are presented as mean ± SD. n is shown in parentheses in the column headings, n is shown in individual cells when reduced due to missing component fatty acids required to calculate ratio-based indices. Overall p denotes the overall difference across age groups. Associations with age treated as a continuous variable are shown as Spearman correlation coefficients (ρ) with 95% confidence intervals (CI) and two-sided p values. Linear regression slopes (per 1-year increase in age) with 95% CI, two-sided p values, and R^2^ are also provided.

In erythrocytes, several indices differed significantly across age groups, including D5D (AA/DGLA) (p < 0.001), D6D proxies based on GLA/LA (p < 0.001) and AA/LA (p < 0.001), ELOVL2-related indices DTA/AA (p = 0.009), DHA/DPA (p < 0.001), DPA/EPA (p < 0.001)), ELOVL5 (p < 0.001), ELOVL6 (p = 0.039), and SCD16 (p < 0.001), whereas D6D (DGLA/LA) and SCD18 showed no overall age-group differences. Post hoc contrasts versus the ≥65-year reference group indicated lower D5D and AA/LA in younger participants (significant in ≤34 and/or 35–44 years old), while elongation-related indices (ELOVL5 and ELOVL2 (DPA/EPA)) were higher in younger groups, consistent with a decline across age categories. Although ELOVL6 showed a significant overall group effect, no individual contrasts versus ≥65 remained significant after multiplicity adjustment, suggesting small, distributed differences. Consistent with the group-based analyses, Spearman correlations for indices in erythrocytes showed positive associations with age for D5D (ρ = 0.162, p < 0.001) and AA/LA (ρ = 0.231, p < 0.001), and inverse associations for elongation-related indices, including ELOVL2 (DTA/AA) (ρ = −0.091, p = 0.003), ELOVL2 (DPA/EPA) (ρ = −0.144, p < 0.001), ELOVL5 (ρ = −0.273, p < 0.001), and ELOVL6 (ρ = −0.111, p < 0.001). The D6D (DGLA/LA) proxy showed only borderline evidence in the Spearman analysis (p = 0.051), although the unadjusted linear slope was significant (p = 0.038).

In plasma, D6D proxies (GLA/LA and DGLA/LA) did not differ across age groups. In contrast, D6D (AA/LA) (p = 0.002), ELOVL2 (DHA/DPA) (p < 0.001), ELOVL2 (DPA/EPA) (p = 0.017), and SCD16 (p = 0.002) showed significant age-group differences. Treating age as a continuous variable, D5D (ρ = 0.157, p < 0.001), AA/LA (ρ = 0.117, p = 0.003), ELOVL2 (DHA/DPA) (ρ = 0.151, p < 0.001), and SCD18 (ρ = 0.085, p = 0.030) were positively correlated with age, whereas ELOVL2 (DPA/EPA) (ρ = −0.117, p = 0.003) and ELOVL6 (ρ = −0.102, p = 0.009) were inversely correlated, SCD16 showed no association. Linear regression results were largely consistent with Spearman analyses, although the association for plasma ELOVL2 (DPA/EPA) did not reach significance in the linear model. Overall, effect sizes were small, with age explaining only a limited proportion of variance.

Analyses in the subset with measurements in both matrices yielded age-related patterns comparable to those in the full dataset. (Supplementary Tables S2A, S2B).

Overall p was derived from Kruskal–Wallis tests across the five age groups. Planned *post hoc* comparisons were limited to contrasts versus the ≥65-year reference group and conducted using Dunn’s test with multiplicity-adjusted p values. Unless otherwise indicated, n shown in cells denotes reduced observations for that index. Spearman correlations are two-sided. Linear regression models include age (years) as an unadjusted continuous predictor; when p and R^2^ appear in the same cell, p is shown above R^2^. Significance codes (*post hoc* vs. ≥ 65): p < 0.05, p < 0.01, p < 0.001 (adjusted).

## 4 Discussion

In our study, we observed age-related differences in blood fatty acid profiles, characterized by relatively higher long-chain n-3 PUFA levels in older adults together with shifts across multiple ratio-based proxy indices of desaturation and elongation in erythrocytes and plasma. These findings are partly consistent with previous reports in which higher EPA and DHA levels were associated with healthier aging or related phenotypes (Sands et al., 2005; Crowe et al., 2008; de Groot et al., 2009). However, evidence remains heterogeneous, for example, Ali et al. reported lower EPA and DHA levels in older long-lived individuals (Ali et al., 2023). Although ALA levels differed little across age groups, higher EPA and DHA levels observed in older adults may reflect differences in diet or supplementation, genetic and lifestyle factors (e.g., smoking), and/or age-related changes in PUFA metabolism. Because circulating long-chain n-3 PUFA largely derive from dietary EPA and DHA, with <10% contributed by endogenous ALA conversion (Burdge and Calder, 2005; Brenna et al., 2009), similar ALA but higher EPA and DHA levels in older adults likely reflect combined dietary and metabolic influences. In this context, the erythrocyte EPA + DHA sum corresponds to the O3I (Harris and Von Schacky, 2004). In our cohort, mean erythrocyte EPA + DHA ranged from 4.7% in the ≤34-year group to 6.0% in the ≥65-year group. On a population level, recent assessments indicate that the average O3I in Germany is approximately 5.8%, reflecting suboptimal long-chain n-3 status relative to the cardioprotective target range of ≥8% proposed in epidemiological studies (Deutsch et al., 2026).

Higher n-3 PUFA status in older adults may be relevant to common chronic diseases, as n-3 PUFA have been linked to reduced susceptibility to cardiovascular and neurodegenerative disorders (Joffre et al., 2020; Oppedisano et al., 2020). Proposed mechanisms include attenuation of inflammation, improvements in lipid profiles and blood pressure effects, and maintenance of neuronal integrity (Siriwardhana et al., 2012; Calder, 2017; Skulas-Ray et al., 2019; Stupin et al., 2019; Wang et al., 2022; Zhang et al., 2022; Sidorkiewicz, 2024). In addition, n-3 PUFA have been associated with a diminished risk of neurodegenerative diseases such as Alzheimer’s and Parkinson’s, and mechanistic pathways have been proposed involving anti-inflammatory actions and maintenance of neuronal integrity (Román et al., 2019; Giacobbe et al., 2020). Age-related changes in nervous system fatty acid composition have been described, and experimental work suggests potential effects on dopaminergic neurons and α-synuclein aggregation (Mota-Martorell et al., 2022; SenGupta et al., 2022).

The synthesis of n-3 and n-6 PUFA involves a competitive process, as both families compete for the same desaturases and elongases. Due to the dietary dominance of n-6 PUFA, n-3 PUFA are often competitively disadvantaged, as both classes rely on the same enzymatic pathways for metabolism (Schmitz and Ecker, 2008). Estimates suggest that the ratio of n-6 to n-3 PUFA intake in Western diets has shifted from approximately 1:1 to around 15–20:1 (Marventano et al., 2015). Consequently, age-related differences in circulating PUFA patterns may primarily reflect differences in dietary intake and/or downstream PUFA handling, rather than a direct increase in endogenous conversion efficiency (Mazereeuw et al., 2012; Mozaffarian and Wu, 2012). We observed consistent age-related differences in plasma and erythrocytes contents of LA and DGLA, consistent with prior reports in plasma phospholipids (de Groot et al., 2009), whereas AA showed less consistent patterns across matrices. As a key structural and signaling lipid involved in membrane function and inflammatory pathways (Abrescia et al., 2020; Di Miceli et al., 2020; Wang et al., 2021), AA may be more tightly regulated due to its rapid conversion into bioactive mediators via cyclooxygenases, lipoxygenases, and cytochrome P450 pathways, as well as degradation by β-oxidation (Funk, 2001; Kroetz and Xu, 2005). These pathways are influenced by inflammatory signals, for example through phospholipase A2 (PLA2)-mediated release of AA from membrane phospholipids (Buczynski et al., 2009; Dennis and Norris, 2015). Increased n-6 PUFA conversion in older adults may contribute to chronic low-grade inflammation, pro-inflammatory monocyte polarization, and elevated risk of chronic disease (Serrano-López and Martín-Antonio, 2021). Experimental studies suggest n-6–rich diets can promote gut dysbiosis and intestinal inflammation (Ghosh et al., 2013), and a meta-analysis of randomized controlled trials reported a 13% higher risk of non-fatal myocardial infarction and coronary heart disease mortality with high n-6 intake (Ramsden et al., 2010).

In the present study, several ratio-based indices exhibited age-related changes, with older participants showing higher D5D and AA/LA values indicating an age-associated shift in n-6 product-to-precursor ratios. Importantly, this observation should not be interpreted independently of diet, supplementation, gut absorption, inflammation, medications, disease status, or genetic factors, all of which can influence circulating fatty acid levels and ratios such as the D5D index (Cormier et al., 2014; Gonzalez-Soto and Mutch, 2021). For example, obesity and insulin resistance can alter DGLA and product-to-precursor ratios, suggesting that these indices may reflect underlying metabolic comorbidities (Tsurutani et al., 2018; Moriyama et al., 2022). The combination of lower LA and DGLA with largely unchanged AA in older adults aligns with age-related metabolic changes and/or variation in determinants of fatty acid ratios. Without direct measurements of relevant gene expression or enzymatic activity, the precise drivers remain uncertain.

In our cohort, elongation-related proxies, particularly ELOVL2 indices, likewise showed consistent age associations. This is notable given that DNA methylation at the ELOVL2 locus is a well-established epigenetic marker of chronological aging (Garagnani et al., 2012). The observed age-dependent shifts in ELOVL2-related ratio proxies across erythrocytes and plasma suggest that these metabolic readouts may reflect, or be linked to, age-related regulation of long-chain PUFA metabolism (Chao and Skowronska-Krawczyk, 2020). More broadly, age-related shifts in elongation-related proxies were further supported by the inverse associations observed for ELOVL6. The significant inverse association of the ELOVL5 proxy in erythrocytes, but not in plasma, underscores that such metabolic patterns can be matrix-specific, likely reflecting differences in lipid pool dynamics and turnover between cellular and circulating compartments. Beyond elongation-related proxies, other indices also showed age-associated trends. Notably, D5D and AA/LA were positively associated with age, indicating a shift in n-6 PUFA metabolism toward downstream products. In contrast, D6D associations were weaker and depended on the product-to-precursor pair used for calculation.

Associations for desaturase proxies were less consistently linked to chronological age. Although SCD16 differed across age groups, no clear monotonic trend was observed with continuous age, and SCD18 associations were weaker and inconsistent. This aligns with the view that SCD indices primarily reflect metabolic status, sensitive to diet, adiposity, and insulin sensitivity, rather than aging *per se* (Vinknes et al., 2013; Domínguez-López et al., 2022). Several factors should be considered when interpreting these findings. First, the indices are product-to-precursor ratios that reflect pathway balance rather than direct enzymatic activity, integrating substrate availability and metabolic demand. Second, inter-individual variation is substantial and influenced by genetic differences in fatty acid metabolism, *FADS* variants show strong associations with circulating PUFA profiles, whereas evidence for ELOVL polymorphisms is limited (Lemaitre et al., 2011; Loukil et al., 2024). Finally, aging-marker signatures may vary across populations, so caution is warranted when generalizing these findings beyond our cohort (De Paoli-Iseppi et al., 2017).

Our study has several limitations. The regional specificity of the cohort may limit generalizability, and only age and sex were consistently available, other covariates—including BMI, laboratory parameters, comorbidities, medications, diet, lifestyle factors, and genetics—were not systematically collected, introducing potential residual confounding. Gut microbiota and related biomarkers were also not assessed. We did not validate desaturase/elongase biology at the protein or gene-expression level, which would strengthen links between ratio-based indices and enzyme activity. Finally, as all participants were clinical patients with variable health status, caution is needed when extrapolating to healthy aging.

Despite these limitations, our findings provide descriptive evidence of age-related differences in blood fatty acid profiles and ratio-based proxies. Given the retrospective design and limited covariate information, these results should be interpreted as observational associations rather than causal mechanisms.

## Data Availability

The original contributions presented in the study are included in the article/Supplementary Material, further inquiries can be directed to the corresponding authors.

## References

[B1] Abrescia P. Treppiccione L. Rossi M. Bergamo P. (2020). Modulatory role of dietary polyunsaturated fatty acids in Nrf2-mediated redox homeostasis. Prog. Lipid Res. 80, 101066. 10.1016/j.plipres.2020.101066 32979455

[B2] Ali S. Aiello A. Zotti T. Accardi G. Cardinale G. Vito P. (2023). Age-associated changes in circulatory fatty acids: new insights on adults and long-lived individuals. Geroscience 45 (2), 781–796. 10.1007/s11357-022-00696-z 36449220 PMC9886696

[B3] Bischoff-Ferrari H. A. Gängler S. Wieczorek M. Belsky D. W. Ryan J. Kressig R. W. (2025). Individual and additive effects of vitamin D, omega-3 and exercise on DNA methylation clocks of biological aging in older adults from the DO-HEALTH trial. Nat. Aging 5 (3), 376–385. 10.1038/s43587-024-00793-y 39900648 PMC11922767

[B4] Borges M. C. Haycock P. Zheng J. Hemani G. Howe L. J. Schmidt A. F. (2022). The impact of fatty acids biosynthesis on the risk of cardiovascular diseases in Europeans and east Asians: a Mendelian randomization study. Hum. Mol. Genet. 31 (23), 4034–4054. 10.1093/hmg/ddac153 35796550 PMC9703943

[B5] Brenna J. T. Salem N. Jr. Sinclair A. J. Cunnane S. C. International Society for the Study of Fatty Acids and Lipids, ISSFAL (2009). Alpha-Linolenic acid supplementation and conversion to n-3 long-chain polyunsaturated fatty acids in humans. Prostagl. Leukot. Essent. Fat. Acids 80 (2-3), 85–91. 10.1016/j.plefa.2009.01.004 19269799

[B6] Buczynski M. W. Dumlao D. S. Dennis E. A. (2009). Thematic review series: proteomics. An integrated omics analysis of eicosanoid biology. J. Lipid Res. 50 (6), 1015–1038. 10.1194/jlr.R900004-JLR200 19244215 PMC2681385

[B7] Burdge G. C. Calder P. C. (2005). Conversion of alpha-linolenic acid to longer-chain polyunsaturated fatty acids in human adults. Reprod. Nutr. Dev. 45 (5), 581–597. 10.1051/rnd:2005047 16188209

[B8] Calder P. C. (2017). Omega-3 fatty acids and inflammatory processes: from molecules to man. Biochem. Soc. Trans. 45 (5), 1105–1115. 10.1042/bst20160474 28900017

[B9] Chao D. L. Skowronska-Krawczyk D. (2020). ELOVL2: not just a biomarker of aging. Transl. Med. Aging 4, 78–80. 10.1016/j.tma.2020.06.004 33043173 PMC7544151

[B10] Cormier H. Rudkowska I. Lemieux S. Couture P. Julien P. Vohl M. C. (2014). Effects of FADS and ELOVL polymorphisms on indexes of desaturase and elongase activities: results from a pre-post fish oil supplementation. Genes Nutr. 9 (6), 437. 10.1007/s12263-014-0437-z 25367143 PMC4235832

[B11] Crowe F. L. Skeaff C. M. Green T. J. Gray A. R. (2008). Serum n-3 long-chain PUFA differ by sex and age in a population-based survey of New Zealand adolescents and adults. Br. J. Nutr. 99 (1), 168–174. 10.1017/s000711450779387x 17678566

[B12] de Carvalho C. Caramujo M. J. (2018). The various roles of fatty acids. Molecules 23 (10), 2583. 10.3390/molecules23102583 30304860 PMC6222795

[B13] de Groot R. H. van Boxtel M. P. Schiepers O. J. Hornstra G. Jolles J. (2009). Age dependence of plasma phospholipid fatty acid levels: potential role of linoleic acid in the age-associated increase in docosahexaenoic acid and eicosapentaenoic acid concentrations. Br. J. Nutr. 102 (7), 1058–1064. 10.1017/s0007114509359103 19402937

[B14] De Paoli-Iseppi R. Deagle B. E. McMahon C. R. Hindell M. A. Dickinson J. L. Jarman S. N. (2017). Measuring animal age with DNA methylation: from humans to wild animals. Front. Genet. 8, 106. 10.3389/fgene.2017.00106 28878806 PMC5572392

[B15] Dennis E. A. Norris P. C. (2015). Eicosanoid storm in infection and inflammation. Nat. Rev. Immunol. 15 (8), 511–523. 10.1038/nri3859 26139350 PMC4606863

[B16] Deutsch T. Harris W. S. Jackson K. H. Hahn A. Schuchardt J. P. (2026). Global comparison of erythrocyte EPA and DHA concentrations in pregnant women. J. Nutr. 156 (2), 101299. 10.1016/j.tjnut.2025.101299 41461259 PMC12975356

[B17] Di Miceli M. Bosch-Bouju C. Layé S. (2020). PUFA and their derivatives in neurotransmission and synapses: a new hallmark of synaptopathies. Proc. Nutr. Soc. 79 (4), 388–403. 10.1017/s0029665120000129 32299516

[B18] Diffenderfer M. R. Rajapakse N. Pham E. He L. Dansinger M. L. Nelson J. R. (2022). Plasma fatty acid profiles: relationships with sex, age, and state-reported heart disease mortality rates in the United States. J. Clin. Lipidol. 16 (2), 184–197. 10.1016/j.jacl.2021.12.005 35120898

[B19] Domínguez-López I. Arancibia-Riveros C. Tresserra-Rimbau A. Castro-Barquero S. Casas R. Vázquez-Ruiz Z. (2022). Relationship between estimated desaturase enzyme activity and metabolic syndrome in a longitudinal study. Front. Nutr. 9, 991277. 10.3389/fnut.2022.991277 36386905 PMC9643862

[B20] Drąg J. Goździalska A. Knapik-Czajka M. Gawędzka A. Gawlik K. Jaśkiewicz J. (2017). Effect of high carbohydrate diet on elongase and desaturase activity and accompanying gene expression in rat's liver. Genes Nutr. 12, 2. 10.1186/s12263-017-0551-9 28138346 PMC5264288

[B21] Dziechciaż M. Filip R. (2014). Biological psychological and social determinants of old age: bio-psycho-social aspects of human aging. Ann. Agric. Environ. Med. 21 (4), 835–838. 10.5604/12321966.1129943 25528930

[B22] Funk C. D. (2001). Prostaglandins and leukotrienes: advances in eicosanoid biology. Science 294 (5548), 1871–1875. 10.1126/science.294.5548.1871 11729303

[B23] Garagnani P. Bacalini M. G. Pirazzini C. Gori D. Giuliani C. Mari D. (2012). Methylation of ELOVL2 gene as a new epigenetic marker of age. Aging Cell 11 (6), 1132–1134. 10.1111/acel.12005 23061750

[B24] Ghosh S. Molcan E. DeCoffe D. Dai C. Gibson D. L. (2013). Diets rich in n-6 PUFA induce intestinal microbial dysbiosis in aged mice. Br. J. Nutr. 110 (3), 515–523. 10.1017/s0007114512005326 23298440

[B25] Giacobbe J. Benoiton B. Zunszain P. Pariante C. M. Borsini A. (2020). The anti-inflammatory role of Omega-3 polyunsaturated fatty acids metabolites in pre-clinical models of psychiatric, neurodegenerative, and neurological disorders. Front. Psychiatry 11, 122. 10.3389/fpsyt.2020.00122 32180741 PMC7059745

[B26] Gonzalez-Soto M. Mutch D. M. (2021). Diet regulation of long-chain PUFA synthesis: role of macronutrients, micronutrients, and polyphenols on Δ-5/Δ-6 desaturases and elongases 2/5. Adv. Nutr. 12 (3), 980–994. 10.1093/advances/nmaa142 33186986 PMC8166571

[B27] Harayama T. Shimizu T. (2020). Roles of polyunsaturated fatty acids, from mediators to membranes. J. Lipid Res. 61 (8), 1150–1160. 10.1194/jlr.R120000800 32487545 PMC7397749

[B28] Harris W. S. Von Schacky C. (2004). The Omega-3 index: a new risk factor for death from coronary heart disease? Prev. Med. 39 (1), 212–220. 10.1016/j.ypmed.2004.02.030 15208005

[B29] Hodson L. Skeaff C. M. Fielding B. A. (2008). Fatty acid composition of adipose tissue and blood in humans and its use as a biomarker of dietary intake. Prog. Lipid Res. 47 (5), 348–380. 10.1016/j.plipres.2008.03.003 18435934

[B30] Hornburg D. Wu S. Moqri M. Zhou X. Contrepois K. Bararpour N. (2023). Dynamic lipidome alterations associated with human health, disease and ageing. Nat. Metab. 5 (9), 1578–1594. 10.1038/s42255-023-00880-1 37697054 PMC10513930

[B31] Horrobin D. F. (1981). Loss of delta-6-desaturase activity as a key factor in aging. Med. Hypotheses 7 (9), 1211–1220. 10.1016/0306-9877(81)90064-5 6270521

[B32] Joffre C. Dinel A. L. Chataigner M. Pallet V. Layé S. (2020). n-3 polyunsaturated fatty acids and their derivates reduce neuroinflammation during aging. Nutrients 12 (3), 647. 10.3390/nu12030647 32121189 PMC7146513

[B33] Kang J. X. Wang J. (2005). A simplified method for analysis of polyunsaturated fatty acids. BMC Biochem. 6, 5. 10.1186/1471-2091-6-5 15790399 PMC1079797

[B34] Kroetz D. L. Xu F. (2005). Regulation and inhibition of arachidonic acid omega-hydroxylases and 20-HETE formation. Annu. Rev. Pharmacol. Toxicol. 45, 413–438. 10.1146/annurev.pharmtox.45.120403.100045 15822183

[B35] Kröger J. Zietemann V. Enzenbach C. Weikert C. Jansen E. H. Döring F. (2011). Erythrocyte membrane phospholipid fatty acids, desaturase activity, and dietary fatty acids in relation to risk of type 2 diabetes in the European Prospective Investigation into Cancer and Nutrition (EPIC)-Potsdam study. Am. J. Clin. Nutr. 93 (1), 127–142. 10.3945/ajcn.110.005447 20980488

[B36] Lattka E. Illig T. Koletzko B. Heinrich J. (2010). Genetic variants of the FADS1 FADS2 gene cluster as related to essential fatty acid metabolism. Curr. Opin. Lipidol. 21 (1), 64–69. 10.1097/MOL.0b013e3283327ca8 19809313

[B37] Lemaitre R. N. Tanaka T. Tang W. Manichaikul A. Foy M. Kabagambe E. K. (2011). Genetic loci associated with plasma phospholipid n-3 fatty acids: a meta-analysis of genome-wide association studies from the CHARGE consortium. PLoS Genet. 7 (7), e1002193. 10.1371/journal.pgen.1002193 21829377 PMC3145614

[B38] Li S. W. Lin K. Ma P. Zhang Z. L. Zhou Y. D. Lu S. Y. (2013). FADS gene polymorphisms confer the risk of coronary artery disease in a Chinese Han population through the altered desaturase activities: based on high-resolution melting analysis. PLoS One 8 (1), e55869. 10.1371/journal.pone.0055869 23383292 PMC3561316

[B39] Loukil I. Mutch D. M. Plourde M. (2024). Genetic association between FADS and ELOVL polymorphisms and the circulating levels of EPA/DHA in humans: a scoping review. Genes Nutr. 19 (1), 11. 10.1186/s12263-024-00747-4 38844860 PMC11157910

[B40] Marventano S. Kolacz P. Castellano S. Galvano F. Buscemi S. Mistretta A. (2015). A review of recent evidence in human studies of n-3 and n-6 PUFA intake on cardiovascular disease, cancer, and depressive disorders: does the ratio really matter? Int. J. Food Sci. Nutr. 66 (6), 611–622. 10.3109/09637486.2015.1077790 26307560

[B41] Mazereeuw G. Lanctôt K. L. Chau S. A. Swardfager W. Herrmann N. (2012). Effects of ω-3 fatty acids on cognitive performance: a meta-analysis. Neurobiol. Aging 33 (7), 1482.e1417. 10.1016/j.neurobiolaging.2011.12.014 22305186

[B42] Moriyama K. Masuda Y. Suzuki N. Yamada C. Kishimoto N. Takashimizu S. (2022). Estimated Elovl6 and delta-5 desaturase activities might represent potential markers for insulin resistance in Japanese adults. J. Diabetes Metab. Disord. 21 (1), 197–207. 10.1007/s40200-021-00958-1 35673485 PMC9167368

[B43] Mota-Martorell N. Andrés-Benito P. Martín-Gari M. Galo-Licona J. D. Sol J. Fernández-Bernal A. (2022). Selective brain regional changes in lipid profile with human aging. Geroscience 44 (2), 763–783. 10.1007/s11357-022-00527-1 35149960 PMC9135931

[B44] Mousavi S. M. Jalilpiran Y. Karimi E. Aune D. Larijani B. Mozaffarian D. (2021). Dietary intake of linoleic acid, its concentrations, and the risk of type 2 diabetes: a systematic review and dose-response meta-analysis of prospective cohort studies. Diabetes Care 44 (9), 2173–2181. 10.2337/dc21-0438 34417277

[B45] Moussa H. Nguile-Makao M. Robitaille K. Guertin M. H. Allaire J. Pelletier J. F. (2019). Omega-3 fatty acids survey in men under active surveillance for prostate cancer: from intake to prostate tissue level. Nutrients 11 (7), 1616. 10.3390/nu11071616 31315273 PMC6683032

[B46] Mozaffarian D. Wu J. H. (2012). (n-3) fatty acids and cardiovascular health: are effects of EPA and DHA shared or complementary? J. Nutr. 142 (3), 614s–625s. 10.3945/jn.111.149633 22279134 PMC3278271

[B47] Nayeem M. A. (2018). Role of oxylipins in cardiovascular diseases. Acta Pharmacol. Sin. 39 (7), 1142–1154. 10.1038/aps.2018.24 29877318 PMC6289399

[B48] Oppedisano F. Macrì R. Gliozzi M. Musolino V. Carresi C. Maiuolo J. (2020). The anti-inflammatory and antioxidant properties of n-3 PUFAs: their role in cardiovascular protection. Biomedicines 8 (9), 306. 10.3390/biomedicines8090306 32854210 PMC7554783

[B49] Ostermann A. I. Müller M. Willenberg I. Schebb N. H. (2014). Determining the fatty acid composition in plasma and tissues as fatty acid methyl esters using gas chromatography – a comparison of different derivatization and extraction procedures. Prostagl. Leukot. Essent. Fat. Acids 91 (6), 235–241. 10.1016/j.plefa.2014.10.002 25458899

[B50] Paparazzo E. Lagani V. Geracitano S. Citrigno L. Aceto M. A. Malvaso A. (2023). An ELOVL2-Based epigenetic clock for forensic age prediction: a systematic review. Int. J. Mol. Sci. 24 (3), 2254. 10.3390/ijms24032254 36768576 PMC9916975

[B51] Parry S. A. Rosqvist F. Peters S. Young R. K. Cornfield T. Dyson P. (2021). The influence of nutritional state on the fatty acid composition of circulating lipid fractions: implications for their use as biomarkers of dietary fat intake. Ups. J. Med. Sci. 126. 10.48101/ujms.v126.7649 34471486 PMC8384057

[B52] Praticò D. Dogné J. M. (2009). Vascular biology of eicosanoids and atherogenesis. Expert Rev. Cardiovasc Ther. 7 (9), 1079–1089. 10.1586/erc.09.91 19764861

[B53] Rabehl M. Wei Z. Leineweber C. G. Enssle J. Rothe M. Jung A. (2024). Effect of FADS1 SNPs rs174546, rs174547 and rs174550 on blood fatty acid profiles and plasma free oxylipins. Front. Nutr. 11, 1356986. 10.3389/fnut.2024.1356986 39021601 PMC11253720

[B54] Ramsden C. E. Hibbeln J. R. Majchrzak S. F. Davis J. M. (2010). n-6 fatty acid-specific and mixed polyunsaturate dietary interventions have different effects on CHD risk: a meta-analysis of randomised controlled trials. Br. J. Nutr. 104 (11), 1586–1600. 10.1017/s0007114510004010 21118617 PMC9422343

[B55] Román G. C. Jackson R. E. Gadhia R. Román A. N. Reis J. (2019). Mediterranean diet: the role of long-chain ω-3 fatty acids in fish; polyphenols in fruits, vegetables, cereals, coffee, tea, cacao and wine; probiotics and vitamins in prevention of stroke, age-related cognitive decline, and Alzheimer disease. Rev. Neurol. Paris. 175 (10), 724–741. 10.1016/j.neurol.2019.08.005 31521398

[B56] Sands S. A. Reid K. J. Windsor S. L. Harris W. S. (2005). The impact of age, body mass index, and fish intake on the EPA and DHA content of human erythrocytes. Lipids 40 (4), 343–347. 10.1007/s11745-006-1392-2 16028715

[B57] Schmitz G. Ecker J. (2008). The opposing effects of n-3 and n-6 fatty acids. Prog. Lipid Res. 47 (2), 147–155. 10.1016/j.plipres.2007.12.004 18198131

[B58] SenGupta T. Lefol Y. Lirussi L. Suaste V. Luders T. Gupta S. (2022). Krill oil protects dopaminergic neurons from age-related degeneration through temporal transcriptome rewiring and suppression of several hallmarks of aging. Aging (Albany NY) 14 (21), 8661–8687. 10.18632/aging.204375 36367773 PMC9699765

[B59] Serrano-López J. Martín-Antonio B. (2021). Inflammaging, an imbalanced immune response that needs to be restored for cancer prevention and treatment in the elderly. Cells 10 (10), 2562. 10.3390/cells10102562 34685542 PMC8533838

[B60] Sherratt S. C. R. Mason R. P. Libby P. Bhatt D. L. (2024). A time to tear Down and a time to mend: the role of eicosanoids in atherosclerosis. Arterioscler. Thromb. Vasc. Biol. 44 (11), 2258–2263. 10.1161/atvbaha.124.319570 39441911 PMC11495529

[B61] Sidorkiewicz M. (2024). The cardioprotective effects of polyunsaturated fatty acids depends on the balance between their Anti- and pro-oxidative properties. Nutrients 16 (22), 3937. 10.3390/nu16223937 39599723 PMC11597422

[B62] Siriwardhana N. Kalupahana N. S. Moustaid-Moussa N. (2012). Health benefits of n-3 polyunsaturated fatty acids: eicosapentaenoic acid and docosahexaenoic acid. Adv. Food Nutr. Res. 65, 211–222. 10.1016/b978-0-12-416003-3.00013-5 22361189

[B63] Skulas-Ray A. C. Wilson P. W. F. Harris W. S. Brinton E. A. Kris-Etherton P. M. Richter C. K. (2019). Omega-3 fatty acids for the management of hypertriglyceridemia: a science advisory from the American heart association. Circulation 140 (12), e673–e691. 10.1161/cir.0000000000000709 31422671

[B64] Stupin M. Kibel A. Stupin A. Selthofer-Relatić K. Matić A. Mihalj M. (2019). The physiological effect of n-3 polyunsaturated fatty acids (n-3 PUFAs) intake and exercise on hemorheology, microvascular function, and physical performance in health and cardiovascular diseases; is there an interaction of exercise and dietary n-3 PUFA intake? Front. Physiol. 10, 1129. 10.3389/fphys.2019.01129 31543828 PMC6728652

[B65] Sunshine H. Iruela-Arispe M. L. (2017). Membrane lipids and cell signaling. Curr. Opin. Lipidol. 28 (5), 408–413. 10.1097/mol.0000000000000443 28692598 PMC5776726

[B66] Svendsen K. Olsen T. Nordstrand Rusvik T. C. Ulven S. M. Holven K. B. Retterstøl K. (2020). Fatty acid profile and estimated desaturase activities in whole blood are associated with metabolic health. Lipids Health Dis. 19 (1), 102. 10.1186/s12944-020-01282-y 32438926 PMC7243306

[B67] Tsurutani Y. Inoue K. Sugisawa C. Saito J. Omura M. Nishikawa T. (2018). Increased serum Dihomo-γ-linolenic acid levels are associated with obesity, body fat accumulation, and insulin resistance in Japanese patients with type 2 diabetes. Intern Med. 57 (20), 2929–2935. 10.2169/internalmedicine.0816-18 29877283 PMC6232036

[B68] Vessby B. Gustafsson I. B. Tengblad S. Berglund L. (2013). Indices of fatty acid desaturase activity in healthy human subjects: effects of different types of dietary fat. Br. J. Nutr. 110 (5), 871–879. 10.1017/s0007114512005934 23414551

[B69] Vinknes K. J. Elshorbagy A. K. Nurk E. Drevon C. A. Gjesdal C. G. Tell G. S. (2013). Plasma stearoyl-CoA desaturase indices: association with lifestyle, diet, and body composition. Obes. (Silver Spring) 21 (3), E294–E302. 10.1002/oby.20011 23404690

[B70] Wang B. Wu L. Chen J. Dong L. Chen C. Wen Z. (2021). Metabolism pathways of arachidonic acids: mechanisms and potential therapeutic targets. Signal Transduct. Target Ther. 6 (1), 94. 10.1038/s41392-020-00443-w 33637672 PMC7910446

[B71] Wang C. Enssle J. Pietzner A. Schmöcker C. Weiland L. Ritter O. (2022). Essential polyunsaturated fatty acids in blood from patients with and without catheter-proven coronary artery disease. Int. J. Mol. Sci. 23 (2), 766. 10.3390/ijms23020766 35054948 PMC8775772

[B72] Warensjö E. Sundström J. Vessby B. Cederholm T. Risérus U. (2008). Markers of dietary fat quality and fatty acid desaturation as predictors of total and cardiovascular mortality: a population-based prospective study. Am. J. Clin. Nutr. 88 (1), 203–209. 10.1093/ajcn/88.1.203 18614742

[B73] Warensjö E. Rosell M. Hellenius M. L. Vessby B. De Faire U. Risérus U. (2009). Associations between estimated fatty acid desaturase activities in serum lipids and adipose tissue in humans: links to obesity and insulin resistance. Lipids Health Dis. 8, 37. 10.1186/1476-511x-8-37 19712485 PMC2746208

[B74] Yuzyuk T. Lozier B. Schwarz E. L. Viau K. Kish-Trier E. De Biase I. (2018). Intra-individual variability of long-chain fatty acids (C12-C24) in plasma and red blood cells. Prostagl. Leukot. Essent. Fat. Acids 135, 30–38. 10.1016/j.plefa.2018.06.006 30103929

[B75] Zhang X. Ritonja J. A. Zhou N. Chen B. E. Li X. (2022). Omega-3 polyunsaturated fatty acids intake and blood pressure: a dose-response meta-analysis of randomized controlled trials. J. Am. Heart Assoc. 11 (11), e025071. 10.1161/jaha.121.025071 35647665 PMC9238708

